# Hair follicle development and related gene and protein expression of skins in Rex rabbits during the first 8 weeks of life

**DOI:** 10.5713/ajas.18.0256

**Published:** 2018-09-13

**Authors:** Zhenyu Wu, Liangzhan Sun, Gongyan Liu, Hongli Liu, Hanzhong Liu, Zhiju Yu, Shuang Xu, Fuchang Li, Yinghe Qin

**Affiliations:** 1 College of Animal Science and Technology, Shandong Agricultural University, Taian, Shandong 271018, China; 2 Shandong Provincial Key Laboratory of Animal Biotechnology and Disease Control and Prevention, Shandong Agricultural University, Taian, Shandong 271018, China; 3 Sichuan Academy of Grassland Sciences, Chengdu, Sichuan 610091, China; 4 College of Animal Science and Technology, China Agricultural University, Beijing 100193, China

**Keywords:** Genes, Proteins, Skin and Hair Follicle, Development, Rex Rabbit

## Abstract

**Objective:**

We aimed to observe hair follicle (HF) development in the dorsal skin and elucidate the expression patterns of genes and proteins related to skin and HF development in Rex rabbits from birth to 8 weeks of age.

**Methods:**

Whole-skin samples were obtained from the backs of Rex rabbits at 0, 2, 4, 6, and 8 weeks of age, the morphological development of primary and secondary HFs was observed, and the gene transcript levels of insulin-like growth factor-I (*IGF-I*), epidermal growth factor (*EGF*), bone morphogenetic protein 2 (*BMP2*), transforming growth factor β-1, 2, and 3 (*TGFβ-1*, *TGFβ-2*, and *TGFβ-3*) were examined using quantitative real-time polymerase chain reaction (PCR). Additionally, Wnt family member 10b (*Wnt10b*) and *β-Catenin* gene and protein expression were examined by quantitative real-time PCR and western blot, respectively.

**Results:**

The results showed significant changes in the differentiation of primary and secondary HFs in Rex rabbits during their first 8 weeks of life. The* IGF-I*, *EGF*, *TGFβ-2*, and *TGFβ-3* transcript levels in the rabbits were significantly lower at 2 weeks of age than at birth and gradually increased thereafter, while the *BMP2* and *TGFβ-1* transcript levels at 2 weeks of age were significantly higher than those at birth and gradually decreased thereafter. *β-Catenin* gene expression was also significantly affected by age, while the *Wnt10b* transcript level was not. However, the Wnt10b and β-catenin protein expression levels were the lowest at 2 and 4 weeks of age.

**Conclusion:**

Our data showed that a series of changes in HFs in dorsal skin occurred during the first 8 weeks. Many genes, such as *IGF-I*, *EGF*, *BMP2*, *TGFβ-1*, *TGFβ-2*, *TGFβ-3*, and *β-Catenin*, participated in this process, and the related proteins *Wnt10b* and *β-Catenin* in skin were also affected by age.

## INTRODUCTION

Animal wool is an important economic resource, and Rex rabbits are famous for their fur and meat. For Rex rabbit production, skin quality is the most important factor. As such, many studies have attempted to identify candidate genes related to animal wool quality. Most of these efforts have focused on humans, sheep and mice. In the present study, we aimed to observe hair follicle (HF) development in the dorsal skin and describe the expression pattern of genes related to skin and HF development in Rex rabbits from birth to 8 weeks of age.

Rabbit hair is an important commercial product generated by Rex rabbits. The HF cycle can typically be divided into three phases: anagen, catagen, and telogen [1]. Similar to the Cashmere goat, the Rex rabbit has a double-coat skin, which contains two distinct types of HFs: primary and secondary HFs [2]. The HFs postnatally enter into the cycle. The HF growth cycle and obviously different sizes of primary and secondary HFs enable the easy differentiation of hair cycle phases and HF types [3].

Many genes, such as epidermal growth factor ( *EGF*), insulin-like growth factor-I (*IGF-I*), bone morphogenetic protein (*BMP*), transforming growth factor β (*TGF-β*), and *Wnt*, have been implicated in HF and skin development. The *EGF* plays an important role in the development of epithelial tissue. The administration of *EGF* to newborn mice delays HF development, decreases the hair growth rate and reduces hair diameter [4,5]. A previous study showed that the physiological levels of *IGF-I* maintain the *in vivo* rates of HF growth *in vitro*, and in the absence of *IGF-I*, HFs enter the catagen stage of the hair growth cycle [6]. Moreover, using semiquantitative polymerase chain reaction (PCR), Little et al [7] showed that *IGF-I* receptor mRNA was downregulated in rat HFs upon the onset of the catagen stage. The *BMP2* has previously been shown to suppress extracellular matrix degradation by inhibiting the expression of matrix metalloproteinase-13 [8].

Members of the TGFβ family play important roles in many essential cellular processes. *TGFβ-1* has been shown to influence proliferation, differentiation, migration and apoptosis, whereas *TGFβ-2* has been implicated in animal immune processes [8,9]. *TGFβs* are membrane-spanning proteins that can be subdivided into three types (*TGFβ-1*, *TGFβ-2*, and *TGFβ-3*) according to their structure and function. *TGFβ-1* and *TGFβ-2* bind to serine/threonine kinase receptors on the plasma membrane and activate Smad molecules as well as additional signaling proteins that work with Smad molecules to regulate gene expression. *TGFβ-3* can affect the binding of *TGFβs* to other receptors [10]. *TGFβ-1* is expressed in nipple cells and the inner root sheath of immature mouse skin. However, *TGFβ-2* and *TGFβ-3* are expressed in only the inner root sheath. In mature mouse skin, *TGFβ-1*, *TGFβ-2*, and *TGFβ-3* are expressed in only the inner root sheath. Thus, the *TGFβ* family likely plays an important role in skin maturation.

*Wnt/β-Catenin* signaling has been implicated in the development of skin and hair as well as the related appendages in the feathers of birds [11]. Wnt family member 10b (*Wnt10b*) promotes hair shaft growth and epithelial differentiation [12, 13]. In the *Wnt/β-Catenin* signaling pathway, *β-Catenin*, which can be translocated to the nucleus, accumulates in the cell plasma. In the nucleus, *β-Catenin* interacts with transcription factor/lymphoid enhancer-binding factor to regulate the expression of background genes [14].

In the present study, we aimed to observed HF development and describe the expression of the genes related to HF development in Rex rabbits from birth to 8 weeks of age.

## MATERIALS AND METHODS

### Experimental rabbits and sample collection

All study procedures were approved by the Shandong Agricultural University Animal Care and Use Committee in accordance with the Guidelines for Experimental Animals established by the Ministry of Science and Technology (Beijing, China). During the trial, the rabbits were housed in a closed and ventilated building at a maximum temperature of 22°C, a minimum temperature of 15°C and a 50% to 60% relative humidity. A 12-h light (6:30 to 18:30) cycle was used. The diets were formulated according to rabbit requirements by using the same formula as De Blas and Wiseman [15]. The feed was pressed into 4-mm pellets using a pellet mill.

Newborn rabbits were 0 weeks old, and whole-skin samples were obtained from the backs of the rabbits at 0, 2, 4, 6, and 8 weeks (6 rabbits per stage and equal ratios for males and females). Two skin samples (1 cm^2^) were collected from the mid-back, and one sample was immediately frozen in liquid nitrogen, transported to the laboratory and stored at −80°C. The other sample was fixed in 4% paraformaldehyde overnight.

### RNA extraction and primer design

**Figure 1:**
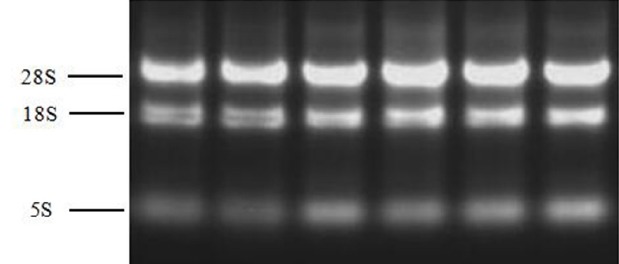
Agarose gel electrophoresis of the extracted total RNA. The 5S, 18S, and 28S RNA were observed, and the brightness ratio between 28S and 18S was calculated as 2.0.

Total RNA was extracted from the samples using the TransZol Up reagent (TransGen Biotech, Beijing, China). RNA integrity was assessed by 1.0% agarose gel electrophoresis. The 28S, 18S, and 5S RNA were observed, and the band brightness ratio between 28S and 18S RNA was calculated as 2.0 (Figure 1). Ultraviolet–visible spectrophotometry (JY04s-3B, Beijing, China) (optical density [OD] at 260 and 280 nm) was used to assess RNA quality and quantity, and the OD 260/280 ratios of the total RNA extract ranged between 1.9 and 2.1. The sequences, GenBank numbers and product lengths for each primer set are shown in Table 1. The primers were designed for exon-intron junctions using Primer 5.0 software or obtained from published literature.

### Real-time polymerase chain reaction

Real-time PCR was conducted using the Applied Biosystems 7500 Real-time PCR System (Applied Biosystems, Carlsbad, CA, USA) and the SYBR Premix Ex Taq Kit (Takara, Dalian, China). The PCR mixtures contained 2 μL of cDNA, 10 μL of SYBR Premix Ex Taq, 0.4 μL of PCR forward primer, 0.4 μL of PCR reverse primer, 0.4 μL of ROX Reference Dye II and 6.8 μL of ddH_2_O. The real-time PCRs were performed at 95°C for 10 s for pre-denaturation, followed by 40 cycles of denaturation at 95°C for 5 s and annealing and extension at 60°C for 40 s. A standard curve was plotted to calculate the efficiency of the real-time PCR primers. Glyceraldehyde-3-phosphate dehydrogenase (GAPDH) was used as the housekeeping gene. Melting curve analysis was performed for all genes and showed that a single amplification product was produced by each reaction. No primer-dimers were generated during the PCR amplification cycles.

### Western blot analysis

The Wnt10b and β-Catenin proteins were analyzed by western blotting. The frozen skin samples were ground with liquid nitrogen and then lysed in 500 μL of precooled Radio Immunoprecipitation Assay buffer (Beyotime, Shanghai, China) with 1 mM phenylmethanesulfonyl fluoride (Beyotime, China). The supernatant was collected after centrifugation at 12,000×g for 10 min at 4°C. The protein concentration was quantified with a BCA Assay Kit (Beyotime, China). An equal amount of protein (40 μg) was separated by sodium dodecyl sulfate-polyacrylamide gel electrophoresis (SDS-PAGE) and transferred onto polyvinylidene fluoride membranes (Millipore, MA, USA). After blocking with triethanolamine-buffered saline containing 5% fat-free milk powder (Beyotime, China) for 1 h at room temperature, the membranes were incubated at 4°C overnight with one of the following primary antibodies: anti-β-catenin from Millipore (Cat.#06-734, USA), anti-Wnt10b from Biorbyt (orb97574, USA), anti-GAPDH from Beyotime (AG019) or anti-Tubulin from Beyotime (AT819). Subsequently, the corresponding secondary antibody (IgG-conjugated horseradish peroxidase) was added, and the samples were incubated for 4 h at 4°C. After washing thrice with Tris-buffered saline containing Tween buffer for 10 min, the signals were developed using an ECL system (Beyotime, China) and visualized by exposing the blots to X-RAY film (Kodak, Rochester, NY, USA). The films were then scanned (HP ScanJet 6100C, Hewlett Packard, Palo alto, CA, USA), and the signal intensity was calibrated by ImageJ 1.43d software (National Institutes of Health, Bethesda, MD, USA).

### Histological examination

The skin samples were fixed with 4% paraformaldehyde and then dehydrated through a graded alcohol series, embedded in paraffin, sectioned at a thickness of 5 μm, and stained with hematoxylin and eosin. The HF development in skin was observed at 100× magnification by using a light microscope.

### Statistical analysis

All data were analyzed using SAS software (SAS version 8e; SAS Institute, Cary. NC, USA). A one-way ANOVA model was used to evaluate the means among the various groups. N = 6 for all mRNA analyses. The data are shown as the means± standard error of the mean. Less than 0.05 p value (p<0.05) was considered as statistically significant.

## RESULTS

### Morphological observation of hair follicle development

**Figure 2:**
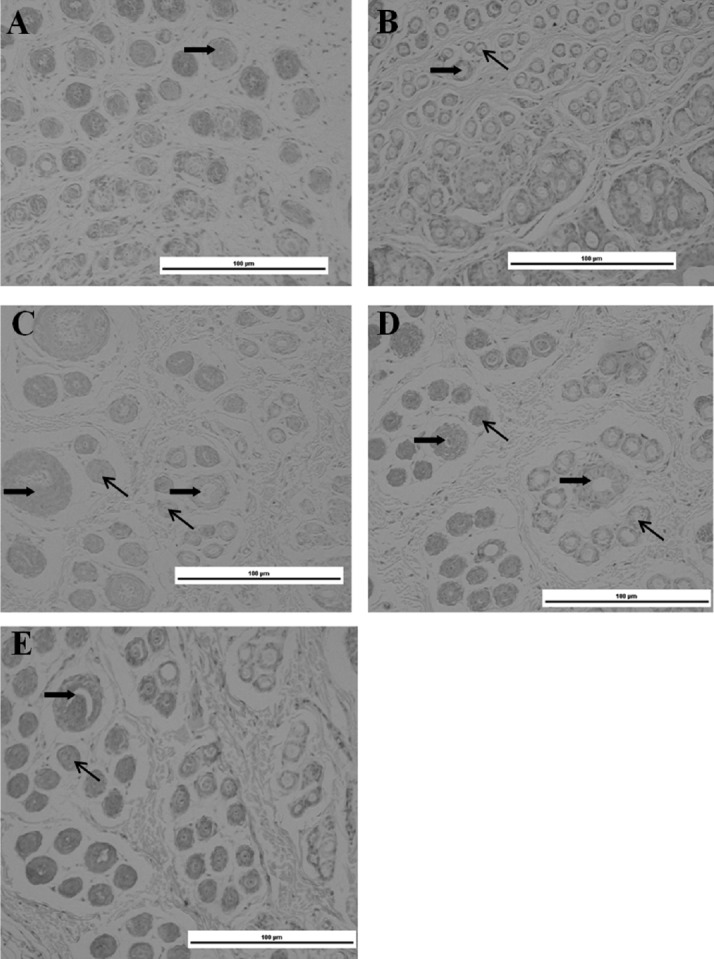
Structure of a vertical slip of skins from 0 to 8 weeks of age. (A) 0 weeks old. (B) 2 weeks old. (C) 4 weeks old. (D) 6 weeks old. (E) 8 weeks old. The skin was sectioned at a thickness of 5 μm and then stained with hematoxyolin and eosin, magnification, 100×; ➡, the primary hair follicle (HF); ➔, the secondary HF.

We observed primary and secondary HF development in dorsal skin. The results showed that the HFs were mainly primary HFs at 0 weeks of age (Figure 2A). At 2 weeks old, the number of differential secondary HFs around the primary HFs reached 2 to 3 (Figure 2B). However, the number of differential secondary HFs around the secondary HFs did not increase, but the diameter of the primary HFs was obviously increased at 4 weeks old (Figure 2C). At 6 and 8 weeks old, the amounts of differential secondary HFs around the primary HFs obviously reached 5 to 13 (Figure 2D).

### Expression profiles of genes related to hair follicle development

**Figure 3:**
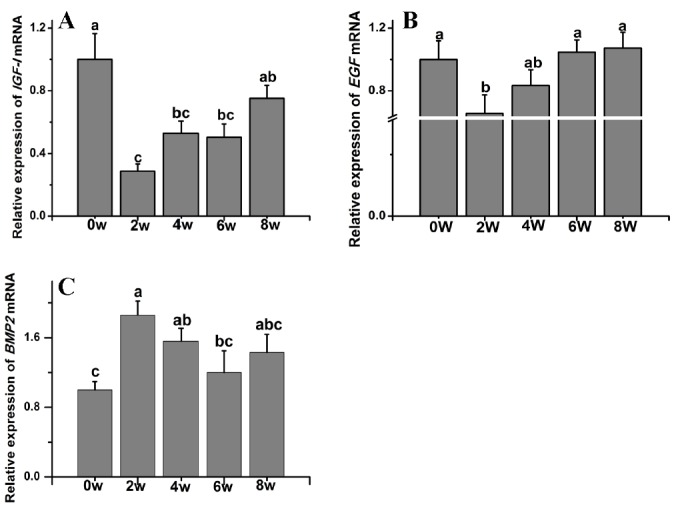
*IGF-I, EGF*, and *BMP2* gene transcript levels in Rex rabbits from birth to 8 weeks old. (A) *IGF-I* transcript levels in Rex rabbits from birth to 8 weeks old. (B) *EGF* transcript levels in Rex rabbits from birth to 8 weeks old. (C) *BMP2* transcript levels in Rex rabbits from birth to 8 weeks old. w, weeks old; *IGF-I*, insulin-like growth I; *EGF*, epidermal growth factor; *BMP2*, bone morphogenetic protein 2. The data are presented as the means±stand error of mean, and n = 6 for each group. ^a,b,c^ Means with different superscripts differ (p<0.05).

*IGF-I* transcripts were detected in skin samples collected from the backs of rabbits at all the tested time points (Figure 3A). The *IGF-I* transcript levels were significantly lower in animals at 2 weeks old than in those at birth (p<0.05). As the rabbits aged, *IGF-I* expression steadily increased until week 8.

The *EGF* transcript levels in rabbits at 2 weeks of age were significantly lower than those at 0, 6, and 8 weeks of age. No significant difference in *EGF* transcript abundance was observed between weeks 2 and 4 (p<0.05) (Figure 3B).

**Figure 4:**
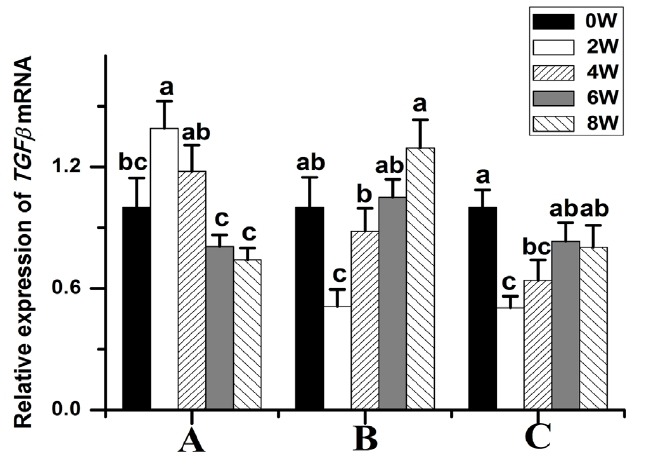
Transforming growth factor β (*TGFβ*) transcript levels in Rex rabbits from birth to 8 weeks old. (A) *TGFβ-1* transcript levels in Rex rabbits from birth to 8 weeks old. (B) *TGFβ-2* transcript levels in Rex rabbits from birth to 8 weeks old. (C) *TGFβ-3* transcript levels in Rex rabbits from birth to 8 weeks old. w, weeks old. The data are presented as the means±stand error of mean, and n = 6 for each group. ^a,b,c^ Means with different superscripts differ (p<0.05).

Compared to those at week 0, the *BMP2* (p<0.05) (Figure 3C) and *TGFβ-1* (p<0.05) (Figure 4A) transcript levels were significantly higher in 2-week-old animals and then gradually decreased thereafter. The *TGFβ-2* transcript levels were significantly lower at week 2. The *TGFβ-2* transcript abundance was highest at week 8 (p<0.05) (Figure 4B). The *TGFβ-3* transcript levels were significantly lower at week 2 compared to those at weeks 0, 6, and 8 (Figure 4C).

**Figure 5:**
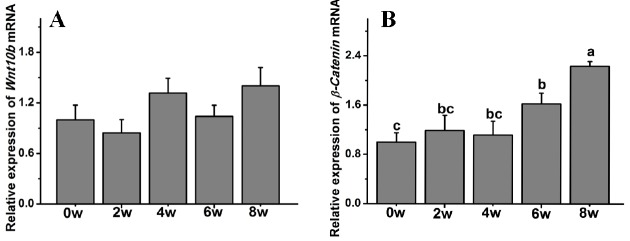
The gene transcript levels of *Wnt10b* and *β-Catenin* in Rex rabbits from birth to 8 weeks old. (A) *Wnt10b* transcript levels in Rex rabbits from birth to 8 weeks old. (B) *β-Catenin* transcript levels in Rex rabbits from birth to 8 weeks old. w, weeks old. The data are presented as the means±stand error of mean, and n = 6 for each group. ^a,b,c^ Means with different superscripts differ (p<0.05).

*β-Catenin* gene expression was also significantly affected by age, while the *Wnt10b* transcript level was not affected in Rex rabbits during the first 8 weeks of postnatal life. The *β-Catenin* transcript levels were significantly increased compared to those at 0 weeks and peaked at 8 weeks old (p<0.05) (Figure 5).

### The patterns of protein expression in dorsal skins

**Figure 6:**
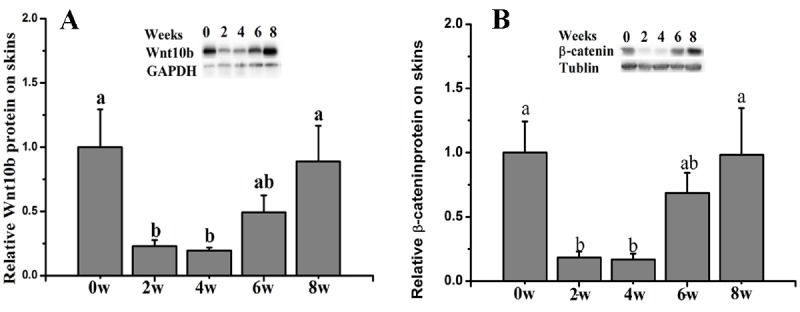
Relative levels of Wnt10b and β-Catenin protein expression in the skins of Rex rabbits from 0 to 8 weeks old. Skin extracts (50 μg protein/sample) were separated by 12% sodium dodecyl sulfate polyacrylamide gel electrophoresis for the determination of Wnt10b, β-Catenin, Tubulin, and GAPDH. The relative Wnt10b expression values were normalized to those of GAPDH, and the relative β-Catenin expression values were normalized to those of Tubulin. The data are presented as the means±stand error of mean. ^a,b^ Means with different superscripts differ (p<0.05), and n = 6 for each group.

The results showed that compared with the control group (0 weeks old), the ratio of β-Catenin protein expression in whole dorsal skin was significantly decreased at 2 and 4 weeks old and gradually increased thereafter (p<0.05) (Figure 6A). Additionally, the Wnt10b protein expression was also significantly decreased at 2 and 4 weeks old and then increased thereafter (p<0.05) (Figure 6b).

## DISCUSSION

Skin growth and HF development are closely related, and many genes have been implicated in these processes. In the present study, we found that HFs have already formed in the back skin of rabbits at 0 weeks old, and genes such as *IGF-I*, *EGF*, *BMP2*, *TGFβ-1*, *TGFβ-2*, *TGFβ-3*, *Wnt10b*, and *β-Catenin* are all expressed in dorsal back skin at 0 weeks old. These results suggest that HF development is already underway at the embryo stage.

There are two distinct types of HFs in the skin of Rex rabbits: primary and secondary HFs [2]. During postnatal development, HFs show cyclic activity with periods of telogen, anagen and catagen expression [16,17]. Many genes, such as *IGF-I*, *EGF*, *BMP*, *TGF-β*, and *Wnt*, have previously been identified as being important in this process. For example, *IGF-I* has a positive effect on the growth of cultured skin cells [6] and affects HF development [18]. The effect of *EGF* on HFs in epithelial tissue differs depending on the hair growth cycle stage [19]. *BMP* signaling can inhibit HF regeneration via the maintenance of HFs in the telogen stage and the prevention of their activation by the advancing regenerative wave [20]. There are at least two distinct *TGF-β* family pathways; one pathway is shared by *TGFβ*, and the other is shared by the *BMP2* and *BMP4* subfamilies. *Wnt/β-Catenin* signaling is also required for the initiation and regeneration of HF development in mice [21]. In the present study, we detected a series of changes in the two types of HFs in Rex rabbits during the first 8 weeks of postnatal life. Moreover, the *IGF-I*, *EGF*, *BMP2*, *TGFβ-1*, *TGFβ-2*, *TGFβ-3*, *Wnt10b*, and *β-Catenin* transcript levels were also significantly changed. These results suggested that the development of HF is closely related to the expression of these genes.

Hair development and the subsequent cycling follow a care fully choreographed rhythm, with cycling being controlled by multiple genes and pathways [22]. Among these pathways, Wnt/β-Catenin signaling has been implicated in the development of skin and hair [11], and this pathway is required for the initiation of HF development [21]. In mice, Wnt10b was shown to promote HF growth *in vitro* and induce HF regeneration via the Wnt/β-Catenin signaling pathway [23,24]. In the present study, we examined Wnt10b and β-Catenin protein and gene expression. The results showed similar Wnt10b and β-Catenin protein expression during HF development in dorsal skin. Both Wnt10b and β-Catenin were significantly decreased in 2- and 4-week-old Rex rabbits. The results also suggested that Wnt/β-Catenin signaling is involved in HF development. However, the transcript levels of Wnt10b and β-Catenin were inconsistent with their protein expression, suggesting that regulation may occur at the translational level. Indeed, a relationship between mRNA and protein expression more intrinsic and complex than a strictly linear relationship may exist [25].

## Figures and Tables

**Table 1:** Primers used for real-time polymerase chain reaction

Gene	GenBank accession number	Primer sequence (5′-3′)	Product size (bp)/Tm (°C)
*GAPDH*	NM_001082253.1	5′-TCACCATCTTCCAGGAGCGA-3′; 5′-CACAATGCCGAAGTGGTCGT-3′	293/60
*IGF-I*	NM_001082026.1	5′-GTGGAGACAGGGGCTTTTATTT-3′ 5′-TGTTGGTAGATGGAGGCTGATA-3′	228/58
*EGF*	XM_008267485.1	5′-AATGCCAACTGCACAAACAC-3 5′-CTGAAATGGCGGAACAGAAT-3	102/56
*BMP2*	NM_001082650.1	5′-TCAAGCGAAACACAAACAGC-3 5′-CCACAATCCAGTCGTTCCAC-3	101/58
*TGFβ-1*	NM_008249704.1	5′-CCGTTTCTTTCGTGGGATAC-3 5′-GGTAAGGGAGGAGGGTCTCA-3	108/60
*TGFβ-2*	NM_001082660.1	5′-GAGAGGAGCGACGAGGAGTA-3 5′-TGAGCCAGAGGGTGTTGTAA-3	108/60
*TGFβ-3*	NM_008272016.1	5′-CACAACCCTCACCTCATCCT-3 5′-CCTGGCGGAAGTCAATGTAG-3	160/60
*Wnt10b*	XM_002711076.2	5′-TGTGCCATCCCTCTTCCTTA-3 5′-GGCTCCACCTCTAACTTCTGC-3	150/60
*β-Catenin*	DQ786777.1	5′-TTCTTGGGACTCTTGTTCAGC-3 5′-CACTTGGCACACCATCATCT-3	122/60

*GAPDH*, glyceraldehyde-3-phosphate dehydrogenase; *IGF-I*, insulin-like growth factor-I; *EGF*, epidermal growth factor; *BMP2*, bone morphogenetic protein 2; TGFβ-1, transforming growth factor β-1; *Wnt10b*, Wnt family member 10b.
